# Complete genome sequence and characterization of the haloacid–degrading *Burkholderia caribensis* MBA4

**DOI:** 10.1186/s40793-015-0109-7

**Published:** 2015-12-01

**Authors:** Yanling Pan, Ka Fai Kong, Jimmy S. H. Tsang

**Affiliations:** Molecular Microbiology Laboratory, School of Biological Sciences, The University of Hong Kong, Hong Kong, SAR China

**Keywords:** *Burkholderia caribensis*, Haloacid degradation, Genome sequence, Dehalogenase, Glycolate operon

## Abstract

*Burkholderia caribensis* MBA4 was isolated from soil for its capability to grow on haloacids. This bacterium has a genome size of 9,482,704 bp. Here we report the genome sequences and annotation, together with characteristics of the genome. The complete genome sequence consists of three replicons, comprising 9056 protein-coding genes and 80 RNA genes. Genes responsible for dehalogenation and uptake of haloacids were arranged as an operon. While dehalogenation of haloacetate would produce glycolate, three glycolate operons were identified. Two of these operons contain an upstream *glcC* regulator gene. It is likely that the expression of one of these operons is responsive to haloacetate. Genes responsible for the metabolism of dehalogenation product of halopropionate were also identified.

## Introduction

Human activities are thought to have great impact on the environment. While the development of industry has greatly improved our living condition, it has also escalates many environmental problems. Pollution has been an issue for a long time. Halogenated compounds have been used indiscriminately with the expansion of industrialization. Many of these compounds are found in the environment as disinfection by-product [1]. Not only do they cause environmental problems they also have deleterious impact on our health [2].

Many bacteria are capable of transforming halogenated compounds and utilize them as carbon and energy sources. These bacteria are distinguished by their encoding enzymes known as dehalogenases which catalyze the breakdown of halogenated compounds through cleavage of the carbon-halogen bond [3]. *Burkholderia caribensis* [4] MBA4 was isolated for its ability to mineralize 2-haloacids [5]. The dehalogenase gene, *deh4a*, together with a downstream permease gene, *deh4p*, form an inducible operon that mediate the transformation and uptake of 2-haloacids, respectively, in MBA4 [6]. The dehalogenase has been purified and characterized [5, 7, 8]. The permease has also been investigated [9]. Moreover, MBA4 possesses a cryptic dehalogenase with a signal peptide [10, 11]. While proteomic analysis of the degradation of chloroacetate by MBA4 has been described, the identities of the differentially expressed proteins were hampered by the lack of a comprehensive protein database [12]. The acquisition of a complete genomic sequence deems necessary. Here we describe the characterization of *B. caribensis*MBA4 and its complete genome sequence and annotation, with an emphasis on genomic features and genes related to degradation of haloacids.

## Organism information

### Classification and features

**Fig. 1:**
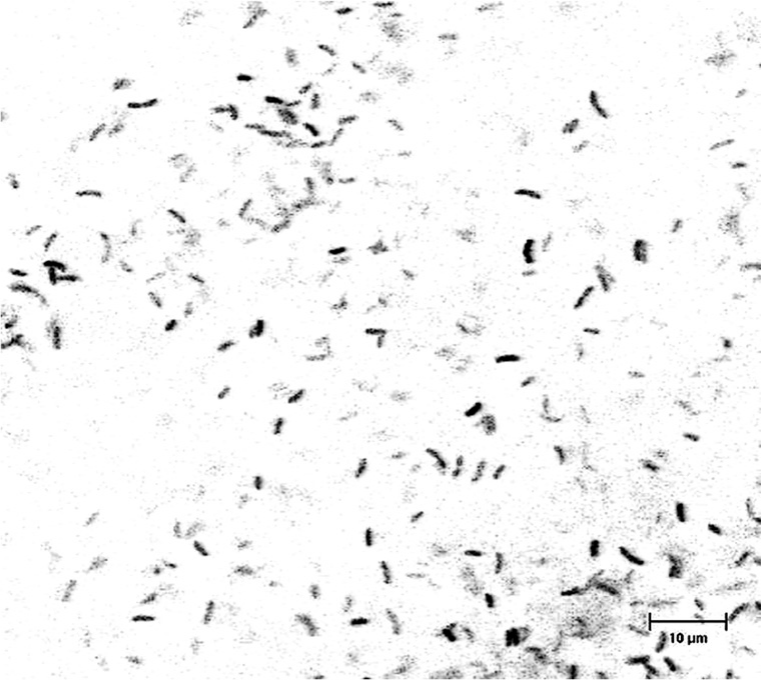
Micrograph of *Burkholderia caribensis* MBA4

**Fig. 2:**
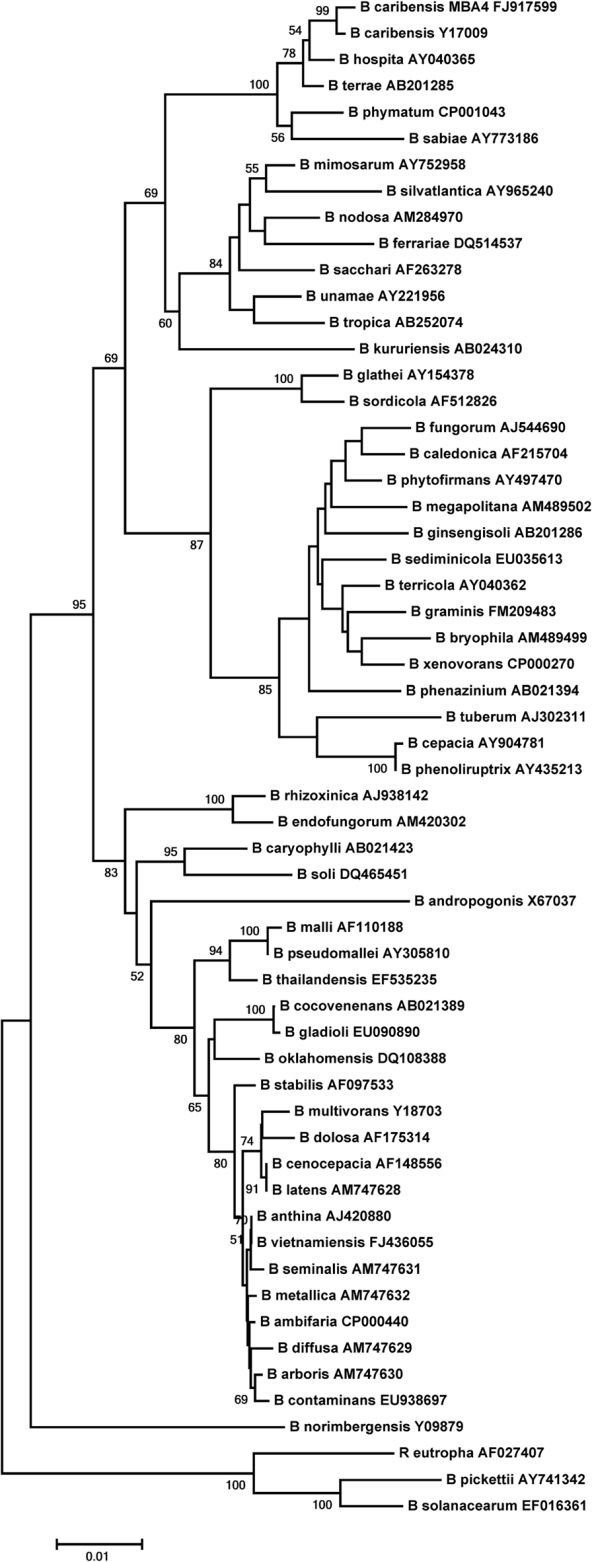
Phylogenetic tree highlighting the relative position of *B. caribensis* MBA4 in the *Burkholderia* genus. The phylogenetic tree was constructed with MEGA6 [34] based on analysis of 16S rDNA sequences. The evolutionary distances were computed using the Maximum Composite Likelihood method [35] and are in the units of the number of base substitutions per site. Numbers at nodes are bootstrap values inferred from 500 replicates. The GenBank accession number and the bacterial species are illustrated

*Burkholderia caribensis*MBA4 (=LMG 28094) is a Gram-negative, motile, rod-shaped bacterium (Fig. 1) in the order *Burkholderiales* [13] and class *Betaproteobacteria* [14]. It grows poorly in traditional Luria-Bertani broth with NaCl but reasonably fast in LB‾ at 30 °C. The general features of this bacterium are shown in Table 1. MBA4 was isolated from forest soil collected from Chiang Mai, Thailand using monobromoacetic acid as an enrichment substrate [5]. In addition to MBA, *B. caribensis*MBA4 is also capable of mineralizing monochloroacetate, 2-monobromopropionate and weakly on 2-monochloropropionate [5]. MBA4 was initially classified as *Pseudomonas cepacia* [5] and subsequently as *Burkholderia cepacia* [15] based on its biochemical and phenotypic features. A polyphasic approach involving phenotypic, genotypic, and phylogenetic analysis was subsequently conducted to have a refined description. API 20NE and BIOLOG GN MicroPlate analyses were performed. These biochemical and substrate assimilation tests show that *B. caribensis*MBA4 failed to reduce nitrates to nitrites, nor from nitrates to nitrogen, incapable of producing indole from tryptophan, cannot acidify glucose and has no arginine dihydrolase nor urease. The bacterium possesses β-galactosidase but no α-glucosidase nor protease. It is able to assimilate glucose, arabinose, mannose, mannitol, N-acetyl-glucosamine, gluconate, caprate, malate and weakly on phenyl-acetate but not on maltose, citrate and adipate. Moreover, MBA4 is able to oxidize Tween-40, Tween-80, N-acetyl-D-galactosamine, adonitol, D-arabitol, D-fructose, L-fucose, m-inositol, L-rhamnose, D-sorbitol, D-trehalose, acetate, methylpyruvate, cis-aconitic acid, formic acid, D-galactonic acid lactone, D-galacturonic acid, D-glucosaminic acid, α-hydroxybutyric acid, β-hydroxybutyric acid, p-hydroxyphenylacetic acid, α-ketobutyric acid, α-ketoglutaric acid, α-ketovaleric acid, D,L-lactic acid, malonic acid, propionic acid, quinic acid, D-saccharic acid, bromosuccinic acid, alaninamide, D,L-alanine, L-alanyl-glycine, L-asparagine, L-aspartic acid, L-glutamic acid, L-histidine, hydroxy-L-proline, L-leucine, L-ornithine, L-phenylalanine, L-proline, L-pyroglutamic acid, L-serine, L-threonine, D,L-carnitine, γ-aminobutyric acid, 2-aminoethanol, glycerol, D,L-α-glycerolphosphate and glucose-6-phosphate and weakly on dextrin, glycogen, psicose, mono-methylsuccinate, succinic acid, succinamic acid, glucuronamide, D-serine and phenylethylamine. While whole cell fatty acid and whole cell protein SDS-PAGE profiles showed that MBA4 is closely related to certain *Burkholderia* species, BOX-PCR fingerprinting analysis [16] showed that the genomic structure of MBA4 is considerably different from other *Burkholderia* species [17]. Phylogenetic analysis using 16S rRNA gene as a marker indicated that MBA4 is most closely related to *B. caribensis*, followed by *B. hospita* [18] and *Burkholderia terrae* [19] (Fig. 2). DNA-DNA hybridization values [20] were determined by the Belgian Coordinated Collections of Microorganisms using *B. caribensis*LMG 18531^T^ and *B. hospita*LMG 20598^T^ as references. Hybridizations were conducted at 50 °C and the values are the mean of four or more tests. A DNA homology value of 74 and 62 % was obtained between MBA4 and LMG 18531^T^, and LMG 20598^T^, respectively [17]. It is thus concluded that MBA4 is a strain of *B. caribensis*.

**Table 1:** Classification and general features of *Burkholderia caribensis* MBA4 according to MIGS recommendations [21]

MIGS ID	Property	Term	Evidence code^a^
	Classification	Domain *Bacteria*	TAS [36]
		Phylum *Proteobacteria*	TAS [37]
		Class *Betaproteobacteria*	TAS [14, 38]
		Order *Burkholderiales*	TAS [13, 38]
		Family *Burkholderiaceae*	TAS [38, 39]
		Genus *Burkholderia*	TAS [15, 40]
		Species *Burkholderia caribensis*	TAS [4]
		Strain: *MBA4*	IDA
	Gram stain	*Negative*	IDA
	Cell shape	*Rod*	IDA
	Motility	*Motile*	IDA
	Sporulation	*Non-sporulating*	IDA
	Temperature range	*30 °C*	IDA
	Optimum temperature	*30 °C*	IDA
	pH range; Optimum	*Not determined*	IDA
	Carbon source	*Haloacids, Pyruvate, Glycolate, Lactate*	IDA
MIGS-6	Habitat	*Soil*	IDA
MIGS-6.3	Salinity	*Not determined*	IDA
MIGS-22	Oxygen requirement	*Aerobic*	IDA
MIGS-15	Biotic relationship	*free-living*	IDA
MIGS-14	Pathogenicity	*Unknown*	IDA
MIGS-4	Geographic location	*Chiang Mai, Thailand*	IDA
MIGS-5	Sample collection	*1984*	IDA
MIGS-4.1	Latitude	*18°47'*	IDA
MIGS-4.2	Longitude	*98°59'*	IDA
MIGS-4.4	Altitude	*310 m*	IDA

^a^ Evidence codes - *IDA* Inferred from Direct Assay, *TAS* Traceable Author Statement (i.e., a direct report exists in the literature), *NAS* Non-traceable Author Statement (i.e., not directly observed for the living, isolated sample, but based on a generally accepted property for the species, or anecdotal evidence). These evidence codes are from the Gene Ontology project [41]

#### Chemotaxonomic data

The whole cell fatty acid profile of *B. caribensis*MBA4 (cells grown on tryptic soy agar) was determined by Department of Biology, The Chinese University of Hong Kong with a Sherlock® Microbial Identification System (Microbial IDentification Inc) using four replicates. The relative abundance for the fatty acids were 14:0 (4.5 %), 16:0 (19.9 %), 16:0 2-OH (1.5 %), 16:0 3-OH (4.2 %), 16:1 2-OH (1.6 %), 17:0 cyclo (12.5 %), 18:0 (1 %), 19:0 ω8c cyclo (6.1 %), summed feature 2 (14:0 3OH, 16:1 iso I, unidentified fatty acid with equivalent chain length value 10.928, 12:0 ALDE, or any of these combination, 6.3 %), summed feature 3 (16:1 ω7c, 15 iso 2OH, or any of their combination, 14.2 %), and summed feature 7 (18:1 ω7c, 18:1 ω9t, 18:1 ω12t, or any of these combination, 26.4 %).

## Genome sequencing information

### Genome project history

The genome of MBA4 was selected for sequencing in order to unravel the genetic background of the bacterium to utilize haloacids. MBA4 has a genome larger than most *Burkholderia* species with a size of more than 9.4 Mbp. Preliminary pulsed-field gel electrophoresis analysis showed that it contains three replicons with sizes of ca. 2.6, 3.5 and 3.7 Mbp (unpublished observations). The high-quality draft genome sequences with annotation were achieved and presented for public access in January 2014. Annotation was updated for the contigs in April 2014. The draft genome sequences was deposited in DDBJ/EMBL/GenBank under the accession number AXDD00000000. The three replicons of the complete genome sequence of MBA4 were finished in October 2015 and have been deposited in GenBank under accession numbers: CP012746, CP012747 and CP012748. Table 2 shows the project information and its association with MIGS version 2.0 compliance [21].

**Table 2:** Project information

MIGS ID	Property	Term
MIGS 31	Finishing quality	Finished
MIGS-28	Libraries used	Four Illumina paired-end libraries, one 454 library, one PacBio 10–20 kb library
MIGS 29	Sequencing platforms	Illumina HisSeq 2000, 454 GS FLX Titanium and PacBio RS II
MIGS 31.2	Fold coverage	850×
MIGS 30	Assemblers	GLC Genomic Workbench 6.0.1, SMRT Analysis v2.3.0 HGAP.2
MIGS 32	Gene calling method	RAST and PGAAP
	Locus Tag	K788
	GenBank ID	CP012746, CP012747, CP012748
	GenBank Date of Release	November, 2015
	GOLD ID	Ga0082378
	BIOPROJECT	PRJNA197459
MIGS 13	Source Material Identifier	MBA4
	Project relevance	Biotechnological, environmental

### Growth conditions and DNA preparation

MBA4 was cultivated in 2 ml LB‾ with shaking at 30 °C. The culture was harvested at late exponential phase with an OD_600_ value of ca. 1.8. The cells were collected by centrifugation at 4000 rpm, 4 °C for 25 min. Genomic DNA was isolated with G-spin™ Genomic DNA extraction kit (iNtRON Biotechnology) according to the manufacturer’s protocol. The yield was about 40 μg and the 260/230 and the 260/280 ratios were 1.9. The concentration of the DNA used for library preparation was 258 ng/μl.

### Genome sequencing and annotation

The genome of MBA4 was sequenced using Illumina HisSeq 2000, 454 GS FLX Titanium and PacBio System. Four sets of Illumina paired-end libraries (insert sizes: 100, 300, 500, and 2000 bp), a set of 454 library and a set of PacBio long read library were constructed. Collectively, the data furnished a coverage of about 850-fold. The raw reads for 500- and 2000-bp paired-end data were obtained from Beijing Genomics Institute while the 100- and 300-bp paired-end data and the 454 reads were obtained from Centre for Genome Sciences (previously Genome Research Centre), The University of Hong Kong. The PacBio long reads were obtained from Groken Bioscience. Bar codes were trimmed and low quality reads were filtered using the commercial software CLC Genomic Workbench 6.0.1 (CLC bio, Aarhus, Denmark). After trimming and filtering, Illumina paired-end and 454 reads were de novo assembled through CLC Genomic Workbench 6.0.1 with default setting. Scaffolds were then generated from the contigs with SSPACE basic 2.0 [22] using information derived from the paired-end reads. De novo assembled transcripts from nine sets of RNA-seq paired-end raw data were mapped to the scaffolds to remove some of the internal gaps and ambiguous bases, and to join the scaffolds together. Standard PCR and Sanger-sequencing technology were employed to fill the gaps inside the scaffolds. Multiplex PCR was used to amplify unknown regions between scaffolds, and some scaffolds were linked after subsequent cloning and sequencing. Clean PacBio reads were assembled by SMRT Analysis v2.3.0 HGAP.2 with pre-assembled high-quality draft genome as reference sequences. Ambiguous base and inserted/deleted regions between PacBio-assembled and preassembled high quality draft sequences were manually corrected using consensus sequences derived from nine sets of transcriptome data.

A draft genome was annotated automatically with the Rapid Annotations using Subsystems Technology server [23–25] and the Prokaryotic Genomes Automatic Annotation Pipeline from NCBI [26]. Subsequent annotation of the complete genome was based on the annotated draft sequences. Minor corrections were conducted manually.

## Genome properties

**Fig. 3:**
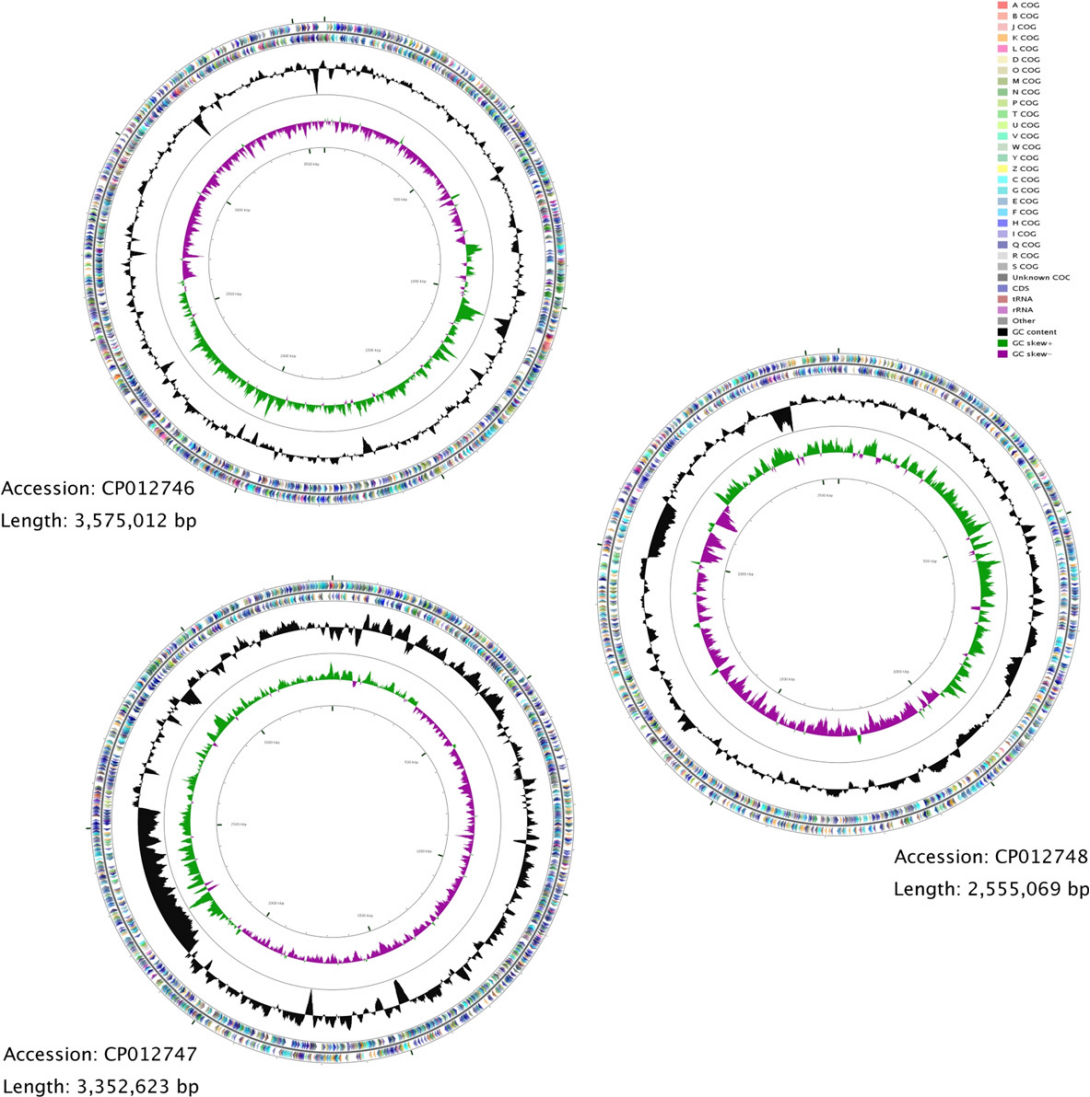
Genome maps of *B. caribensis* MBA4. The outer circle indicates the location of all ORFs. All ORFs were colored according to their COG functional groups. Light venetian red and medium rose colored arrows indicate tRNA and rRNA genes, respectively. GC content is in black and GC skew + and – is in green and fuchsia, respectively. The sizes of the replicons are not drawn to scale

The complete genome is represented by three replicons. The total size of the genome is 9,482,704 bp with a GC content of 62.46 % [27]. A total of 9151 genes were predicted for the genome, including 15 pseudo genes. As for RNA genes, 18 rRNA and 62 tRNA genes were identified. About 80.07 % of the total genes are protein coding with known function while 1729 genes were annotated as hypothetical protein [27]. Among the total, 6596 genes were assigned to COGS. The properties and the statistics of the genome are described in Table 3. The distribution of the genes in COG functional categories [28] is shown in Table 4. Circular genome maps, showed in Fig. 3, were generated using CGview [29] based on ORFs with COG information, tRNA, rRNA and GC content.

**Table 3:** Genome statistics

Attribute	Value	% of Total^a^
Genome size (bp)	9,482,704	100.00
DNA coding (bp)	8,209,808	86.58
DNA G + C (bp)	5,922,869	62.46
DNA scaffolds	3	100.00
Total genes	9151	100.00
Protein coding genes	9056	98.96
RNA genes	80	0.87
Pseudo genes	15	0.16
Genes in internal clusters	Not determined	Not determined
Genes with function prediction	7327	80.07
Genes assigned to COGs	6596	72.84
Genes with Pfam domains	6737	74.39
Genes with signal peptides	824	9.10
Genes with transmembrane helices	2008	22.17
CRISPR repeats	10	

^a^The total is based on either the size of the genome in base pairs or the total number of protein coding genes in the annotated genome

**Table 4:** Number of genes associated with the general COG functional categories

Code	Value	%age^a^	Description
J	215	2.37	Translation, ribosomal structure and biogenesis
A	1	0.01	RNA processing and modification
K	809	8.93	Transcription
L	215	2.37	Replication, recombination and repair
B	4	0.04	Chromatin structure and dynamics
D	44	0.49	Cell cycle control, Cell division, chromosome partitioning
V	65	0.72	Defense mechanisms
T	528	5.83	Signal transduction mechanisms
M	470	5.19	Cell wall/membrane biogenesis
N	159	1.76	Cell motility
U	180	1.99	Intracellular trafficking and secretion
O	224	2.47	Posttranslational modification, protein turnover, chaperones
C	611	6.75	Energy production and conversion
G	625	6.90	Carbohydrate transport and metabolism
E	816	9.01	Amino acid transport and metabolism
F	110	1.21	Nucleotide transport and metabolism
H	246	2.72	Coenzyme transport and metabolism
I	356	3.93	Lipid transport and metabolism
P	359	3.96	Inorganic ion transport and metabolism
Q	253	2.79	Secondary metabolites biosynthesis, transport and catabolism
R	931	10.28	General function prediction only
S	615	6.79	Function unknown
-	2460	27.16	Not in COGs

^a^The total is based on the total number of protein coding genes in the genome

## Insights from the genome sequence

**Fig. 4:**
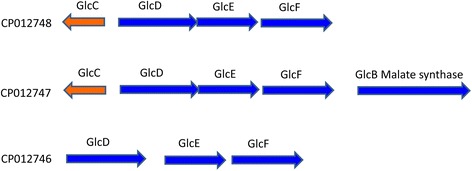
Schematic representation of the genomic organization of three glycolate oxidase genes in *B. caribensis* MBA4. Glycolate oxidase genes comprising *glcDEF* were identified in replicons CP012746, CP012747 and CP012748. In replicons CP012747 and CP012748, a *glcC* regulator gene was also discovered. In replicon CP012747, a *glcB* gene, encoding malate synthase, was found downstream of *glcDEF*

The haloacid utilizing operon, comprising dehalogenase *deh4a* and permease *deh4p* genes, was found in replicon CP012747. Besides *deh4a*, eight other genes are annotated as haloacid dehalogenase or haloacid dehalogenase-like protein for the whole genome. However, in previous studies, when MBA4 was grown in medium containing MCA as the sole carbon and energy source, only Deh4a was detected. A BLASTN analysis showed that these other genes have relatively different nucleotide sequences and which suggested that they are not homologs of *deh4a*. It would be interesting to investigate whether these putative dehalogenases have similar function as Deh4a. When MCA is taken into the cell and processed by Deh4a hydrolytically, glycolate will be produced. Further transformation of glycolate will be mediated by glycolate oxidase, an enzyme that consists of three subunits, viz GlcD, E and F. The genes encoding for glycolate oxidase are clustered as an operon. In MBA4, three glycolate oxidase operons were identified. One of these is located downstream of *deh4a*, in replicon CP012747. This operon has a downstream malate synthase gene, *glcB*, and an upstream regulator gene, *glcC*, in the opposite strand. Another *glcDEF*, also containing an upstream *glcC*, was discovered in replicon CP012748. A third glycolate oxidase operon, located in replicon CP012746, has neither *glcC* nor *glcB* in the neighborhood (Fig. 4). It is apparent that glycolate could be utilized in three ways after transformation to glyoxylate by glycolate oxidase. Whether these three glycolate oxidases are responsible for three different courses awaits further investigation.

For other features of the genome, 612 tandem repeats were found in the genome by Tandem Repeats Finder [30]. There are at least 58 genomic islands being predicted by IslandViewer [31]. On-line CRISPRFinder [32] has identified ten CRISPR regions with one confirmed and nine questionable CRISPRs. Four incomplete and one questionable prophage regions were identified using PHAST [33].

## Conclusions

In this study, we report the complete genome sequence of *Burkholderia caribensis*MBA4 which was isolated for its ability to utilize haloacetates. Examination of genes such as dehalogenases and glycolate oxidases have provided insight on the metabolism of the bacterium in transforming haloacetates for carbon and energy source. Further analysis on genes related to conversion of halopropionate would be fruitful.

## Abbreviations

LB¯: Luria-Bertani broth without NaCl
MBA: Monobromoacetate
MCA: Monochloroacetate

## References

[1] Booth RA, Lester JN (1995). The potential formation of halogenated by-products during peracetic acid treatment of final sewage effluent. Water Res.

[2] Saghir SA, Rozman KK (2003). Kinetics of monochloroacetic acid at subtoxic and toxic doses in rats after single oral and dermal administrations. Toxicol Sci.

[3] Hardman DJ (1991). Biotransformation of halogenated compounds. Crit Rev Biotechnol.

[4] Achouak W, Christen R, Barakat M, Martel MH, Heulin T (1999). *Burkholderia caribensis* sp. nov., an exopolysaccharide-producing bacterium isolated from vertisol microaggregates in Martinique. Int J Syst Bacteriol.

[5] Tsang JSH, Sallis PJ, Bull AT, Hardman DJ (1988). A monobromoacetate dehalogenase from *Pseudomonas cepacia* MBA4. Arch Microbiol.

[6] Yu M, Faan YW, Chung WYK, Tsang JSH (2007). Isolation and characterization of a novel haloacid permease from *Burkholderia cepacia* MBA4. Appl Environ Microbiol.

[7] Pang BCM, Tsang JSH (2001). Mutagenic analysis of the conserved residues in dehalogenase IVa of *Burkholderia cepacia* MBA4. FEMS Microbiol Lett.

[8] Tsang JSH, Pang BCM (2000). Identification of the dimerization domain of dehalogenase IVa of *Burkholderia cepacia* MBA4. Appl Environ Microbiol.

[9] Tse YM, Yu M, Tsang JSH (2009). Topological analysis of a haloacid permease of a Burkholderia sp. bacterium with a PhoA-LacZ reporter. BMC Microbiol.

[10] Tsang JSH, Sam L (1999). Cloning and characterization of a cryptic haloacid dehalogenase from *Burkholderia cepacia* MBA4. J Bacteriol.

[11] Tsang JSH, Sze J (2002). Sec-dependent and Sec-independent translocation of haloacid dehalogenase Chd1 of *Burkholderia cepacia* MBA4 in *Escherichia coli*. FEMS Microbiol Lett.

[12] Kwok SY, Siu AF, Ngai SM, Che CM, Tsang JSH (2007). Proteomic analysis of *Burkholderia cepacia* MBA4 in the degradation of monochloroacetate. Proteomics.

[13] Garrity GM, Bell JA, Lilburn T, Garrity GM, Brenner DJ, Krieg NR, Staley JR (2005). Order I. *Burkholderiales* ord. nov. Bergey’s manual of systematic bacteriology. Second ed. Part C, vol 2.

[14] Garrity GM, Bell JA, Lilburn T, Garrity GM, Brenner DJ, Krieg NR, Staley JR (2005). Class II. *Betaproteobacteria* class. nov. Bergey’s manual of systematic bacteriology. Second ed. Part C, vol 2.

[15] Yabuuchi E, Kosako Y, Oyaizu H, Yano I, Hotta H, Hashimoto Y (1992). Proposal of *Burkholderia* gen. nov. and transfer of seven species of the genus *Pseudomonas* homology group II to the new genus, with the type species *Burkholderia cepacia* (Palleroni and Holmes 1981) comb. nov. Microbiol Immunol.

[16] van Belkum A, Hermans PW (2001). BOX PCR fingerprinting for molecular typing of streptococcus pneumoniae. Methods Mol Med.

[17] Chan YP (2005). Taxonomic analysis of a haloacid degrading *Burkholderia* species MBA4.

[18] Goris J, Dejonghe W, Falsen E, De Clerck E, Geeraerts B, Willems A (2002). Diversity of transconjugants that acquired plasmid pJP4 or pEMT1 after inoculation of a donor strain in the A- and B-horizon of an agricultural soil and description of *Burkholderia hospita* sp. nov. and *Burkholderia terricola* sp. nov. Syst Appl Microbiol.

[19] Yang HC, Im WT, Kim KK, An DS, Lee ST (2006). *Burkholderia terrae* sp. nov., isolated from a forest soil. Int J Syst Evol Microbiol.

[20] Wayne LG, Brenner DJ, Colwell RR, Grimont PAD, Kandler O, Krichevsky MI (1987). Report of the Ad Hoc committee on reconciliation of approaches to bacterial systematics. Int J Syst Evol Microbiol.

[21] Field D, Garrity G, Gray T, Morrison N, Selengut J, Sterk P (2008). The minimum information about a genome sequence (MIGS) specification. Nat Biotechnol.

[22] Boetzer M, Henkel CV, Jansen HJ, Butler D, Pirovano W (2011). Scaffolding pre-assembled contigs using SSPACE. Bioinformatics.

[23] Aziz RK, Bartels D, Best AA, DeJongh M, Disz T, Edwards RA (2008). The RAST Server: rapid annotations using subsystems technology. BMC Genomics.

[24] Overbeek R, Olson R, Pusch GD, Gar JO, Davis JJ, Disz T (2014). The SEED and the Rapid Annotation of Microbial genomes using Subsystems Technology (RAST). Nucleic Acids Res.

[25] Brettin T, Davis JJ, Disz T, Edwards RA, Gerdes S, Olsen GJ (2015). RASTtk: a modular and extensible implementation of the RAST algorithm for building custom annotation pipelines and annotating batches of genomes. Sci Rep.

[26] Angiuoli SV, Gussman A, Klimke W, Cochrane G, Field D, Garrity G (2008). Toward an online repository of Standard Operating Procedures (SOPs) for (meta)genomic annotation. OMICS.

[27] Pan Y, Kong KF, Tsang JSH (2014). Draft genome sequence of the haloacid-degrading *Burkholderia caribensis* strain MBA4. Genome Announcements.

[28] Tatusov RL, Fedorova ND, Jackson JD, Jacobs AR, Kiryutin B, Koonin EV (2003). The COG database: an updated version includes eukaryotes. BMC Bioinformatics.

[29] Grant JR, Stothard P (2008). The CGView server: a comparative genomics tool for circular genomes. Nucleic Acids Res.

[30] Benson G (1999). Tandem repeats finder: a program to analyze DNA sequences. Nucleic Acids Res.

[31] Langille MG, Brinkman FS (2009). IslandViewer: an integrated interface for computational identification and visualization of genomic islands. Bioinformatics.

[32] Grissa I, Vergnaud G, Pourcel C (2007). CRISPRFinder: a web tool to identify clustered regularly interspaced short palindromic repeats. Nucleic Acids Res.

[33] Zhou Y, Liang Y, Lynch KH, Dennis JJ, Wishart DS (2011). PHAST: a fast phage search tool. Nucleic Acids Res.

[34] Tamura K, Stecher G, Peterson D, Filipski A, Kumar S (2013). MEGA6: molecular evolutionary genetics analysis version 6.0. Mol Biol Evol.

[35] Tamura K, Nei M, Kumar S (2004). Prospects for inferring very large phylogenies by using the neighbor-joining method. Proc Natl Acad Sci U S A.

[36] Woese CR, Kandler O, Wheelis ML (1990). Towards a natural system of organisms: proposal for the domains archaea, bacteria, and eucarya. Proc Natl Acad Sci U S A.

[37] Garrity GM, Bell JA, Lilburn T, Garrity GM, Brenner DJ, Krieg NR, Staley JR (2005). Phylum XIV. *Proteobacteria* phyl. nov. Bergey’s manual of systematic bacteriology. Second ed. Part B, vol 2.

[38] Validation List no. 107: List of new names and new combinations previously effectively, but not validly, published. Int J Syst Evol Microbiol. 2006;56:1–6. doi:10.1099/ijs.0.64188-0. 10.1099/ijs.0.64188-0 16403855

[39] Garrity GM, Bell JA, Lilburn T, Garrity GM, Brenner DJ, Krieg NR, Staley JR (2005). Family I. Burkholderiaceae fam. now. Bergey’s manual of systematic bacteriology. Second ed. Part C, vol 2.

[40] Validation of the publication of new names and new combinations previously effectively published outside the IJSB: List No. 45. Int J Syst Evol Microbiol. 1993;43:398–9. doi:10.1099/00207713-43-2-398

[41] Ashburner M, Ball CA, Botstein D, Butler H, Cherry JM, Davis AP (2000). Gene ontology: tool for the unification of biology. The gene ontology consortium. Nat Genet.

